# Genotype heterogeneity of high-risk human papillomavirus infection in Ethiopia

**DOI:** 10.3389/fmicb.2023.1116685

**Published:** 2023-02-10

**Authors:** Ayichew Seyoum, Berhanu Seyoum, Tadesse Gure, Addisu Alemu, Anteneh Belachew, Dessalegn Abeje, Abraham Aseffa, Rawleigh Howe, Andargachew Mulu, Adane Mihret

**Affiliations:** ^1^College of Health and Medical Sciences, Haramaya University, Harar, Ethiopia; ^2^Armauer Hansen Research Institute, Addis Ababa, Ethiopia

**Keywords:** human papillomavirus, cervical cancer, prevalence, genotyping, Ethiopia

## Abstract

Cervical cancer is a vaccine-preventable sexually transmitted disease. In the year 2020, there were an estimated 604,000 new cases and 342,000 deaths worldwide. Although its incidence is global, it is much higher in sub-Saharan African countries. In Ethiopia, there is a scarcity of data about the prevalence of high-risk HPV infection and its association with cytological profiles. Therefore, this study was conducted to fill this information gap. A hospital-based cross-sectional study was conducted from April 26 to August 28, 2021, and enrolled 901 sexually active women. Socio-demographic and other relevant bio-behavioral and clinical data were collected using a standardized questionnaire. Visual inspection with acetic acid [*VIA*] was done as an initial screening method for cervical cancer. The cervical swab was then collected using L-Shaped FLOQSwabs in eNAT nucleic acid preservation and transportation medium. A Pap test was done to determine the cytological profile. Nucleic acid was extracted using STARMag 96 ProPrep Kit on SEEPREP32. A Real-time multiplex assay was performed to amplify and detect the HPV L1 gene used for genotyping. The data were entered into Epi data version 3.1 software and exported to STATA version 14 for analysis. A total of 901 (age range from 30 to 60 years, mean age = 34.8 years, and SD± 5.8) women were screened for cervical cancer using *VIA* and 832 women had a valid co-testing (Pap test and HPV DNA testing) results for further process. The overall prevalence of hr HPV infection was 13.1%. Out of 832 women, 88% of them had normal and 12% had abnormal Pap test results. The proportion of high risk HPV was significantly higher among women with abnormal cytology (*X*^2^ = 688.446, *p* < 0.001) and younger age (*X*^2^ = 15.3408, *p* = 0.018). Among 110 women with hr HPV, 14 genotypes (HPV-16, -18, -31, -33, -35, -39, -45, -51, -52, -56, -58, -59, -66, and -68) were identified while HPV-16, -31, -52, -58, and -35 genotypes were highly prevalent. The high risk HPV infection continues to be a significant public health problem among women 30–35 years old. The presence of high-risk HPV irrespective of genotypes is highly correlated with cervical cell abnormalities. Genotype heterogeneity is observed suggesting the importance of periodic geospatial genotyping surveillance for vaccine effectiveness.

## 1. Introduction

Cervical cancer (CC) is among the cancers caused by HPV infection and the fourth for both incidence and mortality among women (Davies-Oliveira et al., 2021). In 2020, it accounted for an estimated 604,000 new cases and 342,000 deaths (Sung et al., 2021). It was the most commonly diagnosed cancer in 23 countries and was the leading cause of cancer death in 36 countries of which the vast majority of these countries are found in sub-Saharan Africa (Pizzato et al., 2022) including Ethiopia.

The prevalence of hr HPV infection in sub-Saharan African countries is unevenly distributed, ranging from 10.7% (Domfeh et al., 2008) to 97.2% (Sahasrabuddhe et al., 2007). The pooled prevalence of hr HPV in sub-Saharan African countries is 32.3%. Similarly, the genotype distribution of hr HPV varied based on geographical location. For example, in China, HPV-52, -16, -58, -18, and -53 determined as the most prevalent genotypes (Zhang et al., 2020). However, this distribution has a different pattern among African countries. HPV-16, -18, -45, -35, and -33 were the most prevalently identified genotypes among sub-Saharan African countries (Ogembo et al., 2015) while HPV-16, -52, -18, -39, and -31 are the widely distributed in the eastern part of Africa (Seyoum et al., 2022). In Ethiopia, the distribution of hr HPV specifically HPV-16, -52, -18, -58, and -45 is almost similar to the other East African countries (Derbie et al., 2019).

Infection with human papillomavirus (HPV) is the primary cause of cervical cancer (Xing et al., 2021). Around 229 different HPV types have been listed by the International HPV Reference Centre^1^, and this number continues to expand. Among them, about 40 types of HPV can infect the genitals of men and women: the skin of the genitals, the vulva (the area outside the vagina), the anus and the lining of the vagina, the cervix, and the anus. These types can also infect the lining of the mouth and throat (Sendagorta-Cudós et al., 2019).

Genital/mucosal types are of the alpha-PV genus and are classified into oncogenic (high-risk) or non-oncogenic (low-risk) types based on their involvement in malignant lesions. Genotyping of the virus defines by the genetic sequence of the protective outer shell or capsid made of a protein called the Late gene 1 (L1; Soheili et al., 2021). Accordingly, the 15 high-risk (hr) HPVs (HPV-16, -18, -31, -33, -35, -39, -45, -51, -52, -56, -58, -59, -66, -68, -73, and -82) cause dysplasia and cancer. The other 12 are low-risk types (HPV-6, -11, -40, -42, -43, -44, -54, -61, -70, -72, -81, and CP6108), which usually cause low-grade mild dysplasia, genital warts, and respiratory papillomatosis. The remaining three are probable high-risk types (HPV-26, -53, and -66; Asiaf et al., 2014; Doorbar et al., 2015).

Currently, three licensed HPV vaccines constructed using L1 capsid antigens: 9-valent HPV vaccine (Gardasil 9, 9vHPV), quadrivalent HPV vaccine (Gardasil, 4vHPV), and bivalent HPV vaccine (Cervarix, 2vHPV) are available (Gillison et al., 2008; Roden and Stern, 2018). Hence, urgent and bold action is needed to scale up and sustain implementation of the evidence-based interventions to reduce cervical cancer as a public health problem, but such action must be strategic. Since the limited cross-protection offered by the current vaccines, generating scientific data regarding the hr HPV prevalence, genotype distribution, cytological profile and associated factors among the different populations is essential in predicting the efficacy of the current vaccine and devising new vaccine strategy (Senapati et al., 2017). In eastern Ethiopia, there is no data about the prevalence and genotype distribution of the virus. Therefore, this study was conducted to fill this information gap. Therefore, we determined the prevalence of hr HPV infection and cytological profile among sexually active women in Ethiopia.

## 2. Materials and methods

### 2.1. Study settings and design

**Figure 1:**
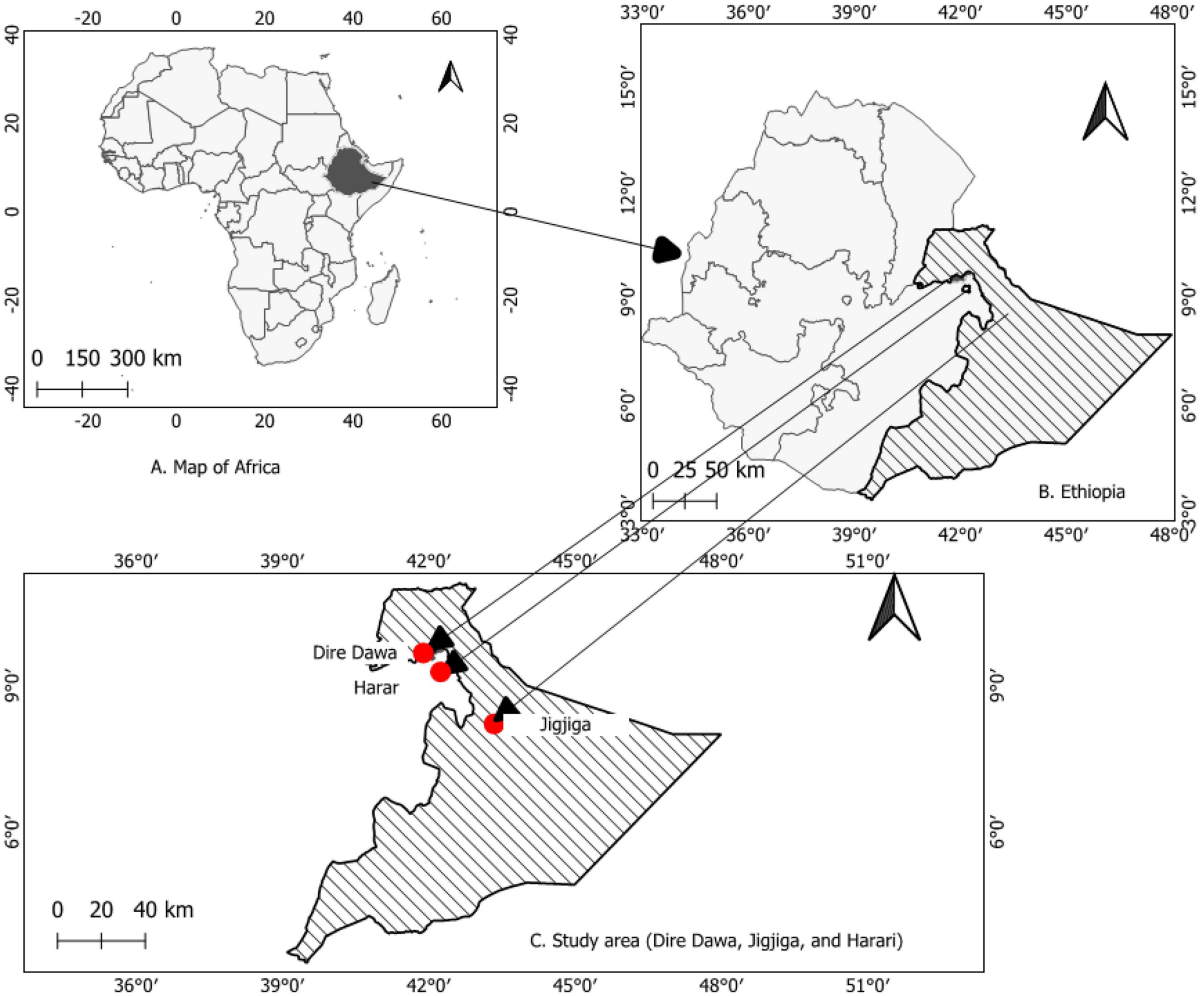
Map of study areas (Extracted using QGIS software).

A health facility-based cross-sectional study was conducted from April 26 to August 28, 2021, in three cities (Harar- Hiwot Fana Specialized University Hospital, Dire Dawa- Dil-Chora Referral Hospital, and Jigjiga- Shiek Hassan Yabare Referral Hospital) in Ethiopia (Figure 1). These health facilities were selected mainly because of their active provision of cervical cancer screening services, and the presence of professionals who perform clinical diagnosis and cytology examinations (gynecologists and pathologists).

### 2.2. Population and eligibility criteria

The source populations of the study were all women who live in eastern Ethiopia and who have started heterosexual intercourse. Women between the ages of 30 and 65 years (WOH, 2021), who have lived in the study area for at least 6 months and who consented to participate in the study were included. Women who had sexual intercourse within 24 h of clinical examination, or who had abundant menstrual bleeding and found it difficult to perform appropriate presumptive screening were excluded. In addition, women with a history of hysterectomy, who were physically or mentally unfit for the interview and pelvic examination for various reasons, were excluded.

### 2.3. Recruitment and sample collection

#### 2.3.1. Demographic and risk factors

Socio-demographic and other relevant bio-behavioral data [such as smoking habits, age at first sexual intercourse, sexual behavior and number of partners, contraceptive use and duration] were collected through a face-to-face interview using pre-designed and pre-tested structured questionnaire. A hospital checklist was used to collect a clinical data [such as number of parity, and history of other sexually transmitted diseases].

#### 2.3.2. Visual inspection with acetic acid

Women who visited the selected hospitals for gynecological problems similar to the HPV virus infection, and met the inclusion criteria were initially screened with *VIA* method for cervical cancer. During *VIA* examination, women with an invisible transition zone were excluded from the study. A sterile plastic spatula was inserted into the vagina to visualize the cervix. Then, 5% acetic acid was applied to the cervix and monitored the changes for 1 min. A sharp, distinct, well-defined, dense (opaque, dull, or oyster white) aceto-white area with or without raised margins define as a positive test (Sankaranarayanan and Wesley, 2003).

#### 2.3.3. Pap smear preparation and result interpretation

After removing the cervical mucus with a cotton swab, the exfoliated ectocervical and endocervical cells were collected using L-shaped Endo/Esocervical eNAT FLOQSwab® (Copan Italia SpA, Brescia, Italy) and make a smear on the slide. The smear was fixed on the slide using ethanol and stained according to standard protocols (Goel et al., 2020). Then, the cytological features of cells were read and results were recorded on standardized forms according to the 2015 Bethesda System which classified women with cytological findings as “normal” or more severe lesions with a positive Pap smear result (abnormal; Nayar and Wilbur, 2015). We excluded all women who had unsatisfactory results from further analysis of the study.

#### 2.3.4. Liquid-based cervical swabs collection and storage

Endocervical and ectocervical cells were collected from the cervical canal using an L-shaped Endo/Ectocervical FLOQSwab® (Copan Italia SpA, Brescia Italy) cytobrush. The brush was then placed into a 2 ml eNAT nucleic acid collection and preservation vial (Copan Italia SpA, Brescia, Italy) for HPV DNA detection and genotyping. The collected cervical cells were transported to Child Health and Mortality Surveillance (CHAMPS) Ethiopia project, Haramaya University, and Armauer Hansen Research Institute (AHRI), Addis Ababa laboratories and stored at -80°C until further processed.

### 2.4. HPV DNA extraction, detection, and genotyping

An aliquot of cervical swab [200 μl] was used to extract nucleic acid using STARMag 96 ProPrep Kit (Seegene, Korea) on SEEPREP32™ (a bead transfer-based nucleic acid extraction instrument for *in vitro* diagnostics) automated Liquid Handling Workstation (Seegene, Korea). The extracted DNA was eluted with 70 μl of elution buffer. Parallel detection and genotyping of HPV was carried out using Anyplex™II HPV HR kit (Seegene, Korea) which can detect and genotype 14 h HPV (HPV-16, -18, -31, -33, -35, -39, -45, -51, -52, -56, -58, -59, -66 and -68). A multiplex Real-time assay was performed to amplify the HPV L1 gene for genotyping and Human housekeeping gene as an endogenous internal control [IC] which can ensure the purification of DNA, verification of PCR reaction, and clarification of cell adequacy from each specimen. CFX96TM Real-time PCR System (Bio-Rad) experiment setup was used for the detection of 14 types of HPV using 5 μl of template DNA in a total volume of 20 μl. The 14 HPV types detection and genotyping were done in five fluorescent channels (FAM, HEX, Cal Red 610, Quasar 670, and Quasar 705), each with individual parameters for target detection and validity; channel 1 HPV-66/-45/-58, channel 2 HPV-51/-59/-16, channel 3 HPV-33/-39/-52, channel 4 HPV-35/-18 and, channel 5 HPV-56/-68/-31.

### 2.5. Statistical methods

The completeness of the collected data is checked before being entered into the database. The data were then cleaned and coded and entered into Epi data version 3.1 software and exported to STATA version 14 for analysis. Frequencies, proportions, and summary statistics were used to describe the study population with relevant variables. A binary logistic regression model was used to identify factors associated with HPV infection and odds ratio with 95% CI was used to assess the degree of association. The *p* value < 0.05 was considered a statistically significant association and variables with *p* < 0.25 were tested for multivariable logistic regression.

### 2.6. Ethical considerations

The study was conducted according to the Helsinki Declaration and Ethiopian research regulations. Both, the Institutional Health Research Ethics Review Committee (IHRERC) of the College of Health and Medical Sciences, Haramaya University, Ethiopia (Reference Number: IHRERC/212/2020) and the Armauer Hansen Research Institute Ethics Committee (Reference Number: PO/20/20) approved the protocol.

## 3. Results

### 3.1. Sociodemographic characteristics

In this study, a total of 901 women (age range from 30 to 60 years, mean age = 34.8 years, and SD = ±5.8) were initially screened using *VIA* screening method. The majorities were urban residents (86.9%) and married (87.1%), while more than half of the study participants were unemployed (65.3%), unable to read and write (54.2%), and over 18 years of age at the time of their first marriage (68.4%) and first sexual intercourse (66.8%; Table 1).

**Table 1:** Socio-demographic characteristics of women who participated in the study.

Variable	Category	Residence (*n* = 901)		
		Urban, *N* (%)	Rural, *N* (%)	Total, *N* (%)
Age (in years)	At 30	269 (34.3)	50 (43.1)	319 (35.4)
	31–35	269 (34.3)	33 (28.4)	302 (33.5)
	36–40	156 (19.9)	23 (19.8)	179 (19.9)
	41–45	48 (6.1)	4 (3.4)	52 (5.8)
	46–50	29 (3.7)	3 (2.6)	32 (3.5)
	51–55	5 (0.6)	1 (0.9)	6 (0.7)
	56–65	9 (1.1)	2 (1.7)	11 (1.2)
Marital status	Married	681 (86.7)	104 (89.7)	785 (87.1)
	Single/ Never married	17 (2.2)	1 (0.7)	18 (2)
	Widowed	27 (3.4)	3 (2.6)	30 (3.3)
	Divorced	57 (7.3)	7 (6)	64 (7.1)
	Separated	3 (0.4)	1 (0.9)	4 (0.4)
Occupational status	Full time employee	180 (22.9)	17 (14.7)	197 (21.9)
	Part-time employee	83 (10.6)	4 (3.4)	87 (9.7)
	Unemployed	503 (64.1)	85 (73.3)	588 (65.3)
	Student	6 (0.8)	2 (1.7)	8 (0.9)
	Retired	2 (0.2)	3 (2.6)	5 (0.5)
	Other	11 (1.4)	5 (4.3)	16 (1.8)
Current Educational status	Unable to read and write	410 (52.2)	78 (67.2)	488 (54.2)
	Elementary/1–8 grades	201 (25.6)	23 (19.8)	224 (24.9)
	High school/9–12 grades	78 (9.9)	6 (5.2)	84 (9.3)
	Tertiary level	96 (12.2)	9 (7.8)	105 (11.6)
Age at first marriage (in years)	<15	35 (4.5)	6 (5.2)	41 (4.5)
	15–17	189 (24.1)	55 (47.4)	244 (27.1)
	≥18	561 (71.5)	55 (47.4)	616 (68.4)
Age at first sexual intercourse (in years)	<15	41 (5.2)	9 (7.8)	50 (5.5)
	15–17	199 (25.3)	50 (43.1)	249 (27.6)
	≥18	545 (69.4)	57 (49.1)	602 (66.8)

### 3.2. Cytological profile of the study participants

**Figure 2:**
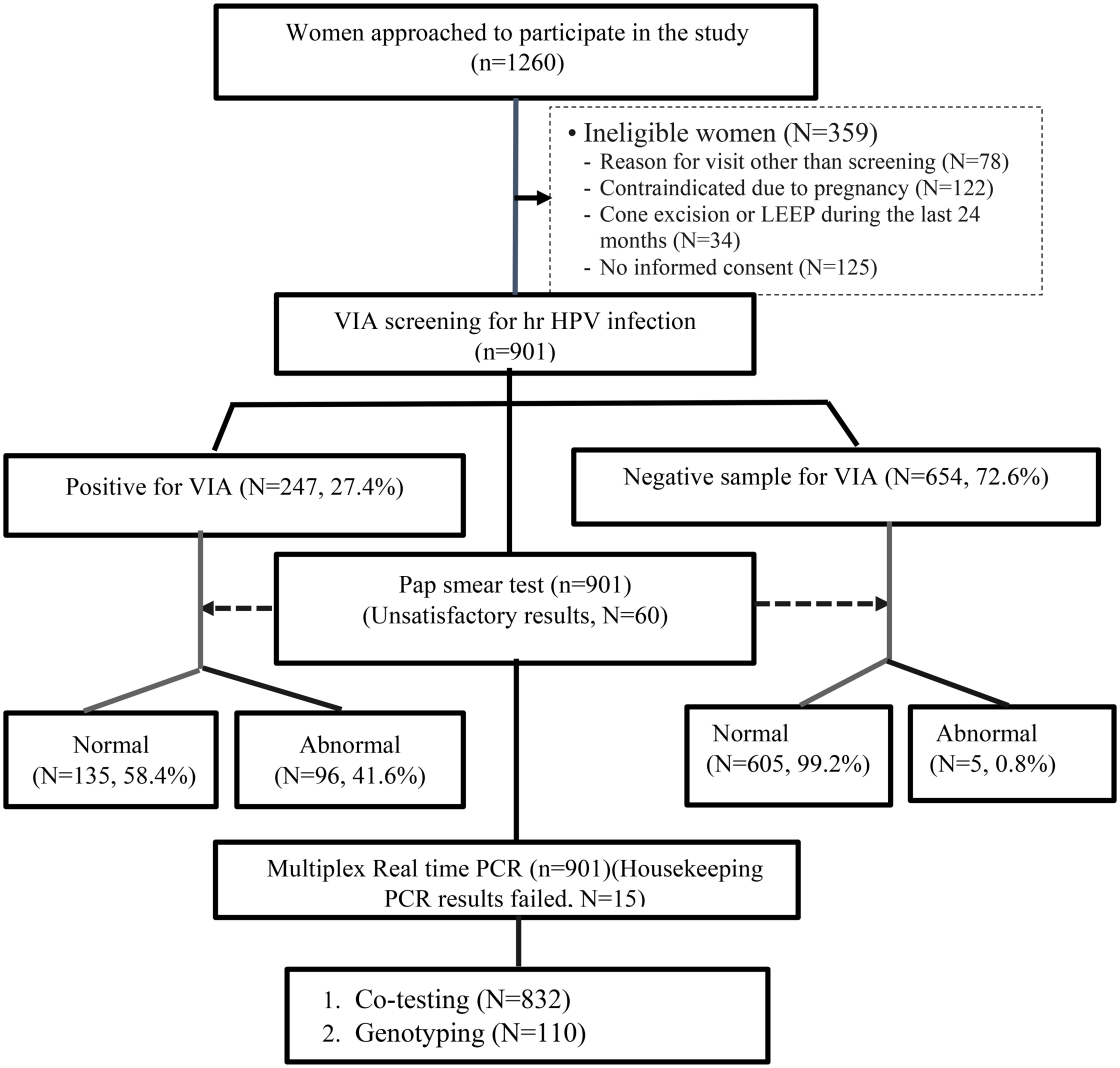
Flowchart for the liquid-based cytology and HPV DNA testing performed on the study population.

Among 901 women who had *VIA* screening and Pap smear test, 654 (72.6%) women had negative and the remaining 247 (27.4%) positive *VIA* results. But, during the Pap smear test, 60 (6.7%) women were excluded from the study due to “unsatisfactory/unreliability test result for the evaluation of cervical epithelial cell abnormalities.” Therefore, we included only 841 women who had normal (740, 88%) and abnormal (101, 12%) Pap test results for further analysis. Out of 101 women with abnormal cytology results, 98 (97%) had Low-grade squamous intraepithelial lesions (LSIL), and 3 (3%) had High-grade squamous intraepithelial lesions (HSIL; Figure 2).

### 3.3. Prevalence of hr HPV based on cytology outcome

Among 901 women who were diagnosed with *VIA*, 15 women had invalid PCR results due to inadequate specimen collection, processing, or the presence of inhibitors and were excluded from further analysis. However, of the remaining 886 women’s samples, the hr HPV was detected on 110 (12.4%). There was also a significant difference in the proportion of hr HPV detection between the *VIA*-positive and negative women (97.3% vs. 2.7%, *p* < 0.001; Table 2).

**Table 2:** Prevalence of hr HPV infection based on cytology outcome among Ethiopian women.

Cytology tests	Category	HPV DNA test			*p* value
		Not detected, *N* (%)	Detected, *N* (%)	Total, *N* (%)	
*VIA* (*n* = 886)	Negative	641 (82.6)	3 (2.7)*	644 (72.7)	<0.001
	Positive	135 (17.4)	107 (97.3)*	242 (27.3)	
Pap test (*n* = 832)	Normal	719 (99.4)	13 (11.9)*	732 (88)	<0.001
	Abnormal	4 (0.6)	96 (88.1)*	100 (12)	

*Statistically significant association.

Similarly, out of 901 women who underwent co-testing (Pap and HPV DNA test), 54 women had unsatisfactory Pap test results and 9 women had invalid HPV DNA testing results. Further, the results of 6 women for both the Pap test and HPV DNA testing were invalid. Therefore, the co-testing results of 832 women were used for further analysis. The overall proportion of hr HPV infection was 13.1%, and the rate of hr HPV detection was significantly higher in women with an abnormal Pap test result compared to women with a normal Pap test (88.1% vs. 11.9%, *p* < 0.001; Table 2).

**Figure 3:**
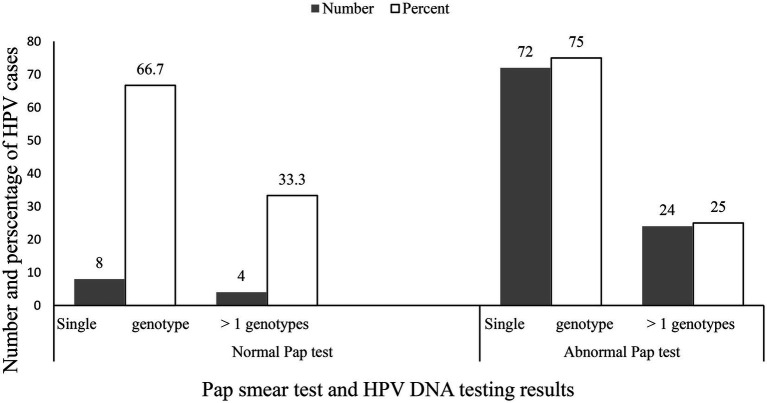
A single and co-existed genotypes of HPV within different Pap test results.

This study also revealed that the detection rate of hr HPV infection was significantly higher in women with LSIL cytology results (86.2% vs. 0.4%, *p* < 0.001; Table 3). In addition, 8 (66.7%) of the 12 women who had a normal Pap smear result were affected by a single genotype while out of 96 who had abnormal Pap smear result, 72 (75%) women were affected by a single genotype, and the remaining 24 (25%) women were affected by more than 1 genotype (Figure 3).

**Table 3:** Prevalence of hr HPV infection based on Pap test results in Ethiopian women.

Pap test results (*n* = 832)		PCR results (*n* = 832)		Total, *N* (%)
		Detected, *N* (%)	Not detected, *N* (%)	
	Normal	13 (11.9)	719 (99.4)	732 (88,0)
	Low grade SIL	94 (86.2)*	3 (0.4)*	97 (11.7)
	High grade SIL	2 (1.8)	1 (0.1)	3 (0.4)

*Statistically significant association.

**Figure 4:**
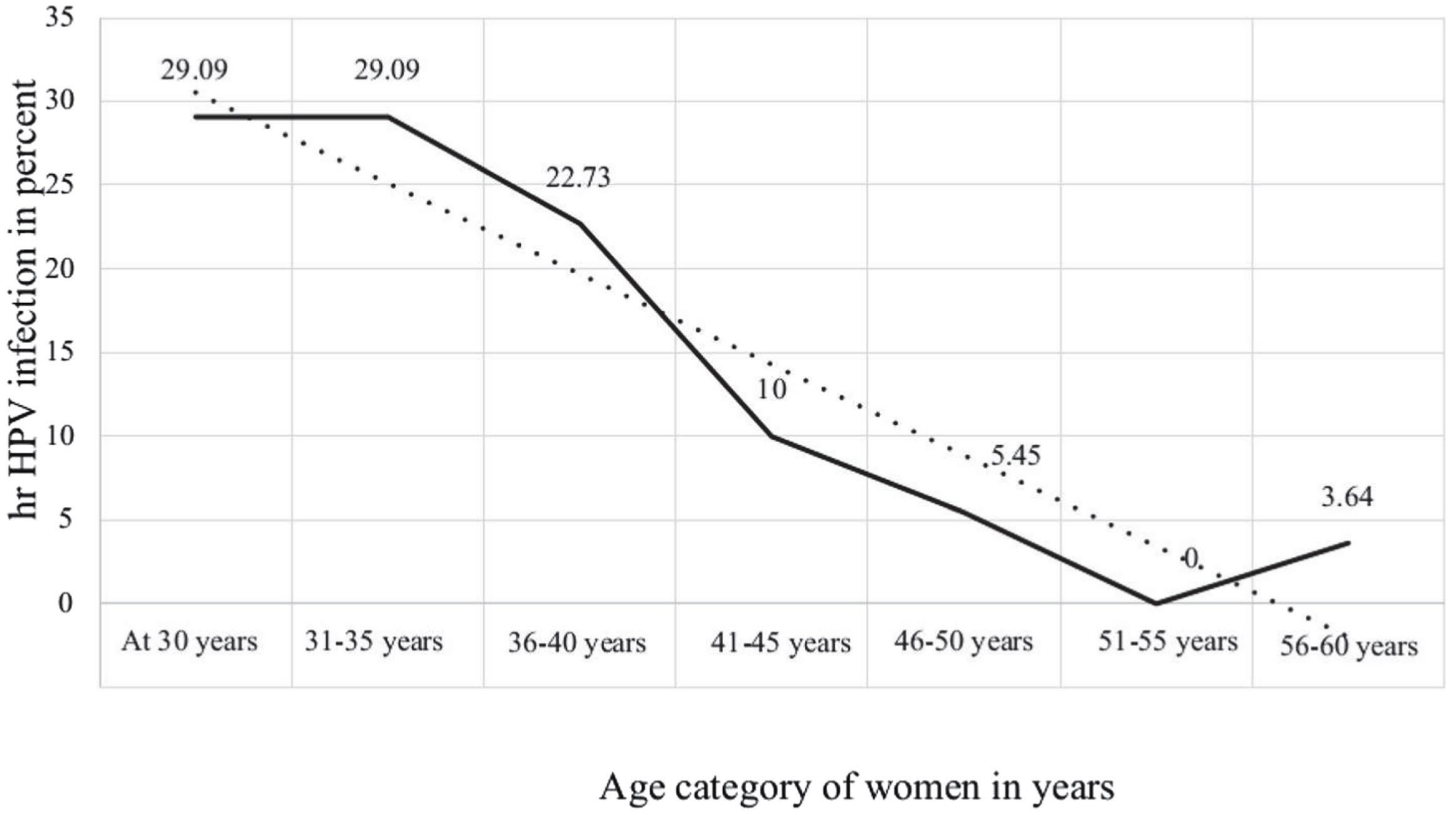
Age-specific prevalence of hr HPV infection among women in Ethiopia.

Among 886 women aged 30 to 60 with valid HPV DNA results, the highest proportion of hr HPV infection (29.1%) was observed in women aged 30 to 35 years. In addition to that, the proportion of hr HPV infection decreased as the age of the women increased, and statistically, it has a significant association (*X*^2^ = 15.3408, *p* = 0.018) (Figure 4).

### 3.4. Type-specific prevalence of hr HPV infection

**Figure 5:**
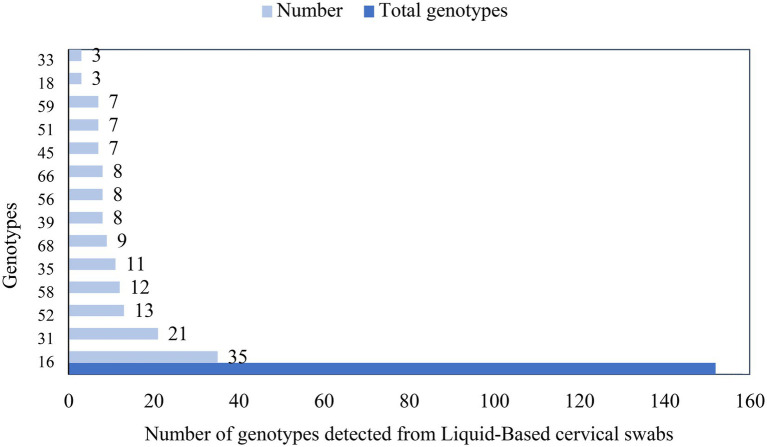
Genotypes and frequency of hr HPV among women in Ethiopia.

In the study, 14 genotypes were identified among 110 women whose hr HPV DNA was detected. Among these, 80 (72.7%) women had a single genotype and the remaining 30 (27.3%) women had more than one (multiple) genotype. In addition, Hr HPV-16 (31.8%), -31(19.1%), -52(11.8%), 58(10.9%), and -35(10%) were the most frequently detected genotypes (Figure 5).

### 3.5. Factors associated with hr HPV infection on the multivariate logistic regression model

We first explored the main factors associated with hr HPV infection in this study using a binary logistic regression model. Then, we selected only associated factors with a *p* value <0.25 and entered them into the multivariate logistic regression model.

Accordingly, depending on their potential risk with different sex partners the odds of getting the hr HPV infection among women with single marital status is higher than married women (AOR = 8.9, 95% CI: 2.05–38.64, *p* = 0.004). Similarly, the odds of getting the hr HPV infection among women who had more than one sexual partner is higher than women who had a single sexual partner (AOR = 7.14, 95%CI: 3.08–16.54, *p* < 0.001).

The crude and adjusted effects of selected covariates obtained from logistic regression are summarized in Table 4.

**Table 4:** Shows the associated factors for hr HPV infection on the multivariate logistic regression model.

Variable name	Category	HPV DNA testing (*n* = 886)		COR (95% CI)	AOR (95% CI)	*p* value
		Detected, *N* (%)	Not detected, *N* (%)			
Age (in years)	At 30	32(29.09)	283 (36.47)	*R*	*R*	
	31–35	32(29.09)	263 (33.89)	1.08 (0.64–1.81)	0.68 (0.26–1.68)	0.390
	36–40	25 (22.73)	151 (19.46)	1.46 (0.84–2.56)	0.65 (0.25–1.70)	0.380
	41–45	11(10.00)	41 (5.28)	2.37 (1.11–5.07)	0.89 (0.28–2.79)	0.845
	46–50	6 (5.5)	26 (3.4)	2.04 (0.78–5.33)	1.07 (0.24–4.83)	0.925
	51–55	–	6 (0.8)	–	–	–
	56–60	4 (3.6)	6 (0.8)	5.89 (1.58–22.00)	2.39 (0.18–31.62)	0.509
Residence	Urban	100 (90.91)	670 (86.34)	*R*	*R*	
	Rural	10 (9.09)	106 (13.66)	0.63 (0.32–1.25)	0.39 (0.08–1.93)	0.252
Marital status	Married	82 (74.55)	688 (88.66)	*R*	*R*	
	Single/Never married	9 (8.18)	9 (1.16)	8.39 (3.24–21.73)	8.90 (2.05–38.64)	0.004*
	Widowed	5 (4.55)	25 (3.22)	1.68 (0.62–4.50)	1.05 (0.24–4.61)	0.949
	Divorced	13 (11.82)	51 (6.57)	2.14 (1.11–4.09)	1.27 (0.48–3.41)	0.630
	Separated	1 (0.91)	3 (0.39)	2.80 (0.29–27.20)	2.31 (0.05–103.33)	0.666
Educational status	Unable to read and write	44 (40)	434 (55.9)	*R*	*R*	
	Elementary/1–8 grades	35 (31.8)	189 (24.4)	1.83 (1.13–2.94)	0.85 (0.34–2.16)	0.738
	High school/9–12 grades	14 (12.73)	69 (8.9)	2.00 (1.04–3.84)	1.93 (0.62–5.96)	0.253
	Tertiary level (Diploma and above)	17 (15.5)	84 (10.8)	1.99 (1.09–3.66)	1.22 (0.41–3.63)	0.720
Age at first marriage	<15 years	11 (10)	30 (3.9)	*R*	*R*	
	15–17 years	29 (26.4)	210 (27.1)	0.38 (0.17–0.83)	5.98 (0.20–176.22)	0.300
	≥18 years	70 (63.6)	536 (69.1)	0.36 (0.17–0.74)	2.37 (0.09–61.17)	0.604
Age at first sexual intercourse	<15 years	13 (11.8)	37(4.8)	*R*	*R*	
	15–17 years	28(25.5)	215(27.7)	0.37 (0.18–0.78)	0.12 (0.01–2.69)	0.185
	≥18 years	69(62.7)	524(67.5)	0.36 (0.19–0.74)	0.19 (0.01–3.96)	0.289
Sexual partners	Singe	573 (73.8)	21 (19.1)	*R*	*R*	
	Multiple	203 (26.2)	89 (80.9)	11.96 (7.24–19.76)	7.14 (3.08–16.54)	<0.001*
Long-term use of oral contraceptive (*n* = 272)**	Yes	48 (85.1)	198 (91.7)	*R*	*R*	
	No	8 (14.3)	18 (8.3)	1.83 (0.75–4.47)	1.77 (0.64–4.89)	0.271

*Presence of statistically significant association (*p* < 0.05). **≥10 years. *R* = 1. AOR, adjusted odds ratio. CI, confidence interval. COR, crude odds ratio. R, reference.

## 4. Discussion

This study is the first hospital-based study conducted at the molecular level to determine the molecular epidemiology of hr HPV infection among sexually active women in eastern Ethiopia. The presence of high prevalence and genotype heterogeneity of hr HPV as a cause of multiple HPV infections indicates a major public health problem that requires greater attention. Additionally, it showed that the detection rate of the virus has a direct correlation with abnormal cytology.

The overall prevalence of hr HPV infection was determined to be 13.1% which is low compared to previous studies in various African countries as high as 95% for example in Benin (Tounkara et al., 2020), 83.2% in Ethiopia (Wolday et al., 2018), 76.3% in South Africa (Ebrahim et al., 2016), 57.7% in Kenya (Menon et al., 2016), 53% in Zimbabwe (Marembo et al., 2019), 48.7% in Mozambique (Omar et al., 2017), 46.2% in Swaziland (Ginindza et al., 2017), and 41.5% in Burkina Faso (Ogembo et al., 2015) and genotype heterogeneity with 14 genotypes of hr HPV were identified. The low prevalence in the current study might be explained by the differences in the occupational and health status of the study participants. The current study was conducted on women with a different type of occupations and gynecological problems (from asymptomatic to invasive stage) with normal or abnormal cytological status. This proportional inclusion of women allowed might help us to identify the high level of genotype heterogeneity of hr HPV among women. However, various studies we used for comparison were conducted on female sex workers and people living with HIV (PLWHIV). As a result, the prevalence of HPV infection was 2–3 times higher among these segments of the population.

In contrast, the hr HPV infection in this study was higher compared to the research findings of 9.4% in Iran (Malary et al., 2016) and 12% in the Gambia (Camara et al., 2018), with comparable results of 13.1 and 13.7% were found in Tunisia (Ardhaoui et al., 2016) and Ethiopia (Ali et al., 2019). This comparable result may be due to the fact that the women included in the studies we used for comparison were sexually active women, which is similar to the participants in this study. Therefore, it is important to consider the demographic and health-related aspects of a population segment in order to make appropriate comparisons and take targeted interventions.

The prevalence of hr HPV infection among women with normal cytology was 11.9% and it was very close to the global average of 11.7% (Scott-Wittenborn and Fakhry, 2021) and 12.8% in Northern Africa, but it was low compared to the average of 57.3% in the southern, 42.2% in eastern, and 27.8% in western Africa regions (Ogembo et al., 2015). In the current study, HPV-16, -31, -52, -39, and -45 were the top five HPV types infecting women with normal cytology. This distribution was inconsistent with the global; HPV-16, -52, -31, and -53 (Bruni et al., 2019) and eastern African countries; HPV-16, -52, -18, -51, and -58 (Ogembo et al., 2015). Contrary to normal cytology, the highest prevalence of hr HPV infection (88.1%) was determined in women with abnormal cytology. The result of the current study is inconsistent with study findings conducted in the Middle East and North Africa (MENA) where the pooled prevalence was 54% (Obeid et al., 2020). Furthermore, in the current study, HPV-16 and -31 genotypes were found to be the main cause of lesions in women with abnormal cytology. This was inconsistent with the results of a meta-analysis in East African countries where HPV-16 and -52 were the main causes of lesions with ASCUS, LSIL, and HSIL cytological results. Whereas HPV-16 and -18 were the predominant HPV genotypes found in women with ICC. Among the possible reasons for this inconsistency could be related to bio-behavioral characteristics including cultural differences in age at first intercourse, lifetime number of sexual partners, and current smoking status (Lin et al., 2015). The distribution of HPV genotypes is spatially inconsistent across continents, countries, and even within a single country. This lack of uniform distribution makes the identification of HPV genotypes in every locality critical to implement vaccine-based disease preventive measures. In agreement with this conclusion, this study has a significant role in informing the HPV genotypes among women in eastern Ethiopia.

The current study also found that the hr HPV infection rate decreases as the age of the study participants’ increase. The highest prevalence of hr HPV infection (29.09%) was observed in women between 30 and 35 years of age, while the prevalence of infection was comparatively lower in women over 40 years of age. The age-specific prevalence of the disease in the current study is consistent with the results of previous studies (Smith et al., 2008; Bekos et al., 2018). This is likely due to the interaction between the natural history of the disease and the genotypes that cause the lesion. Molecular studies on HPV have suggested that patient age and HPV genotypes are independent factors influencing the regression and progression rates of cervical lesions (Ho et al., 1998; Bosch and Harper, 2006). Studies show that young women generally have higher spontaneous recovery (Cox et al., 2003; Moscicki et al., 2010).

Along with women’s age, the other strongest factor influencing the natural history of the disease is the presence of hr HPV (particularly HPV-16 and -18 genotypes) infection (Moscicki et al., 1998). These two genotypes increase the risk of persistent infection. In addition to these two main factors, smoking (Appleby et al., 2006), multiparity, and long-term use of oral contraceptives can double or triple the risk for progression to high-grade lesions or cervical cancer in HPV-infected women (International Collaboration of Epidemiological Studies of Cervical Cancer, 2006). If this study had been conducted using a longitudinal study design, it would have been possible to determine the viral persistence/ progression rate and assess the impact of hr HPV. However, we were forced to use a cross-sectional study design because of insufficient financial resources to conduct a longitudinal study design. This can be considered a limitation of this study.

## 5. Conclusion

The hr HPV infection continues to be a major public health problem among women of 30–35 years old. Although the prevalence was high in younger women, the age-specific HPV infection prevalence declines as the age increase. The presence of hr HPV irrespective of genotypes is highly correlated with cervical cell abnormalities. Genotype heterogeneity is observed suggesting the importance of periodic geospatial genotyping surveillance for vaccine effectiveness.

## Data availability statement

The original contributions presented in the study are included in the article, further inquiries can be directed to the corresponding author.

## Ethics statement

The studies involving human participants were reviewed and approved by Institutional Health Research Ethics Review Committee (IHRERC) of the College of Health and Medical Sciences, Haramaya University, Ethiopia and the Armauer Hansen Research Institute Ethics Committee. The patients/participants provided their written informed consent to participate in this study.

## Author contributions

AS, AdM, AnM, BS, RH, and AbA participated in proposal development, data collection, laboratory works, data analysis, and manuscript writing. TG conducted clinical examination, sample collection, and supervision of midwives during sample collection. AdA and AB participated in the cytological examination. DA participated in nucleic acid extraction, laboratory protocol review, HPV detection, and HPV genotyping. All authors contributed to the article and approved the submitted version.

## Conflict of interest

The authors declare that the research was conducted in the absence of any commercial or financial relationships that could be construed as a potential conflict of interest.

## Publisher’s note

All claims expressed in this article are solely those of the authors and do not necessarily represent those of their affiliated organizations, or those of the publisher, the editors and the reviewers. Any product that may be evaluated in this article, or claim that may be made by its manufacturer, is not guaranteed or endorsed by the publisher.

## Footnotes

^1^www.hpvcenter.se, on 26/08/2022.
