# Corrigendum: Genotype heterogeneity of high-risk human papillomavirus infection in Ethiopia

**DOI:** 10.3389/fmicb.2023.1178530

**Published:** 2023-03-23

**Authors:** Ayichew Seyoum, Berhanu Seyoum, Tadesse Gure, Addisu Alemu, Anteneh Belachew, Dessalegn Abeje, Abraham Aseffa, Rawleigh Howe, Andargachew Mulu, Adane Mihret

**Affiliations:** ^1^College of Health and Medical Sciences, Haramaya University, Harar, Ethiopia; ^2^Armauer Hansen Research Institute, Addis Ababa, Ethiopia

**Keywords:** human papillomavirus, cervical cancer, prevalence, genotyping, Ethiopia

In the original article, there was an error in affiliation 3. Instead of “TDR, the Special Program for Research and Training in Tropical Diseases, WHO, Geneva, Switzerland”, it should be “Armauer Hansen Research Institute, Addis Ababa, Ethiopia”, which is listed as affiliation 2.

In the original article, there was a mistake in Figure 4, “Age category of women in years” and the age category on the x-axis in Figure 4. “Age-specific prevalence of hr HPV infection among women in Ethiopia” was not correctly indicated as published.

**Figure 4:**
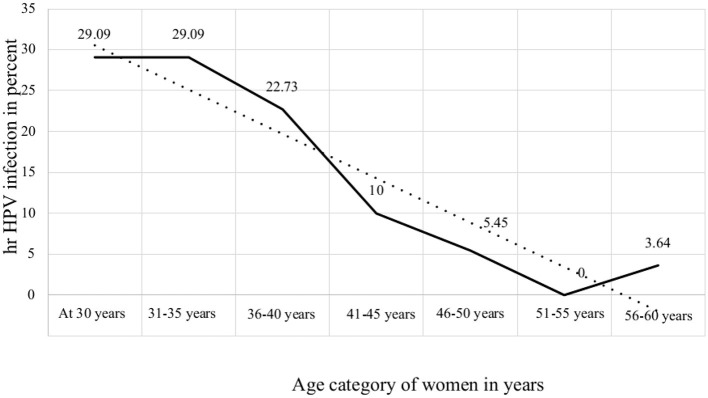
Age-specific prevalence of hr HPV infection among women in Ethiopia.

The corrected label of the x-axis of Figure 4 is “Age category of women in years” and the age category of women is listed, “at 30 years, 31–35 years, 36–40 years, 41–45 years, 46–50 years, 51–55 years, 56–60 years,” as shown in the figure.

The authors apologize for these errors and state that this does not change the scientific conclusions of the article in any way. The original article has been updated.

